# Chemokine (C‐C Motif) Receptor‐Like 2 is not essential for lung injury, lung inflammation, or airway hyperresponsiveness induced by acute exposure to ozone

**DOI:** 10.14814/phy2.13545

**Published:** 2017-12-15

**Authors:** Farhan Malik, Kevin R. Cromar, Constance L. Atkins, Roger E. Price, William T. Jackson, Saad R. Siddiqui, Chantal Y. Spencer, Nicholas C. Mitchell, Ikram U. Haque, Richard A. Johnston

**Affiliations:** ^1^ Division of Critical Care Medicine Department of Pediatrics McGovern Medical School at The University of Texas Health Science Center at Houston Houston Texas; ^2^ Marron Institute of Urban Management New York University New York New York; ^3^ Division of Pulmonary Medicine Department of Pediatrics McGovern Medical School at The University of Texas Health Science Center at Houston Houston Texas; ^4^ Comparative Pathology Laboratory Center for Comparative Medicine Baylor College of Medicine Houston Texas; ^5^ Section of Pediatric Pulmonology Department of Pediatrics Baylor College of Medicine Houston Texas; ^6^ Department of Integrative Biology and Pharmacology McGovern Medical School at The University of Texas Health Science Center at Houston Houston Texas

**Keywords:** chemerin, Cmklr1, CXCR2, Gpr1, methacholine, osteopontin

## Abstract

Inhalation of ozone (O_3_), a gaseous air pollutant, causes lung injury, lung inflammation, and airway hyperresponsiveness. Macrophages, mast cells, and neutrophils contribute to one or more of these sequelae induced by O_3_. Furthermore, each of these aforementioned cells express chemokine (C‐C motif) receptor‐like 2 (Ccrl2), an atypical chemokine receptor that facilitates leukocyte chemotaxis. Given that Ccrl2 is expressed by cells essential to the development of O_3_‐induced lung pathology and that chemerin, a Ccrl2 ligand, is increased in bronchoalveolar lavage fluid (BALF) by O_3_, we hypothesized that Ccrl2 contributes to the development of lung injury, lung inflammation, and airway hyperresponsiveness induced by O_3_. To that end, we measured indices of lung injury (BALF protein, BALF epithelial cells, and bronchiolar epithelial injury), lung inflammation (BALF cytokines and BALF leukocytes), and airway responsiveness to acetyl‐*β*‐methylcholine chloride (respiratory system resistance) in wild‐type and mice genetically deficient in Ccrl2 (Ccrl2‐deficient mice) 4 and/or 24 hours following cessation of acute exposure to either filtered room air (air) or O_3_. In air‐exposed mice, BALF chemerin was greater in Ccrl2‐deficient as compared to wild‐type mice. O_3_ increased BALF chemerin in mice of both genotypes, yet following O_3_ exposure, BALF chemerin was greater in Ccrl2‐deficient as compared to wild‐type mice. O_3_ increased indices of lung injury, lung inflammation, and airway responsiveness. Nevertheless, no indices were different between genotypes following O_3_ exposure. In conclusion, we demonstrate that Ccrl2 modulates chemerin levels in the epithelial lining fluid of the lungs but does not contribute to the development of O_3_‐induced lung pathology.

## Introduction

Chemotactic cytokines, more commonly known as chemokines, direct leukocyte migration following interaction with seven‐transmembrane domain receptors that are part of the chemokine receptor family (Bachelerie et al. 2014). Chemokine (C‐C motif) receptor‐like 2 (Ccrl2) is a member of this family and is expressed by a variety of cells, including astrocytes, B lymphocytes, dendritic cells, endothelial cells, macrophages, mast cells, microglia, and neutrophils (Shimada et al. 1998; Brouwer et al. 2004; Galligan et al. 2004; Oostendorp et al. 2004; Hartmann et al. 2008; Zabel et al. 2008; Otero et al. 2010; Monnier et al. 2012; Bachelerie et al. 2014; Del Prete et al. 2017; Monaghan 2017). While the majority of receptors within the chemokine receptor family initiate leukocyte trafficking *via* G protein‐dependent signaling (Bachelerie et al. 2014), Ccrl2 is incapable of activating G proteins (De Henau et al. 2016), yet Ccrl2 has the ability to facilitate leukocyte chemotaxis (Zabel et al. 2008; Otero et al. 2010; Monnier et al. 2012; Del Prete et al. 2017). Because Ccrl2 influences leukocyte migration in the absence of G protein signaling, Ccrl2 is subclassified as an atypical chemokine receptor within the chemokine receptor family (Bachelerie et al. 2014). Atypical chemokine receptors are characterized by the absence of a canonical DRYLAIV motif within the second intracellular loop of the seven‐transmembrane domain receptor (Graham et al. 2012), and because Ccrl2 lacks the DRYLAIV motif that is necessary to bind G proteins (Bachelerie et al. 2014; Aken et al. 2017), Ccrl2 cannot initiate G protein signaling (De Henau et al. 2016). Zabel et al. (2008) identified chemerin, a nonchemokine chemoattractant for macrophages, natural killer cells, and plasmacytoid dendritic cells, as an endogenous ligand for Ccrl2 (Wittamer et al. 2003; Zabel et al. 2005; Parolini et al. 2007; Bondue et al. 2011), and in support of the classification of Ccrl2 as an atypical chemokine receptor, chemerin does not activate G protein signaling when binding Ccrl2 (De Henau et al. 2016). In addition to Ccrl2, chemerin is a ligand for chemokine‐like receptor 1 (Cmklr1) and G protein‐coupled receptor 1 (Gpr1) (Bondue et al. 2011), and both Cmklr1 and Gpr1 can activate G proteins (Rourke et al. 2015). With that said, the precise mechanisms by which Ccrl2 influences cell migration remain unresolved. Nevertheless, Zabel et al. (2008) hypothesize that Ccrl2 mediates leukocyte migration by presenting chemerin to Cmklr1 which is essential for chemerin‐induced chemotaxis of macrophages, natural killer cells, and plasmacytoid dendritic cells (Wittamer et al. 2003; Zabel et al. 2005; Parolini et al. 2007). Furthermore, from the observation that Ccrl2 increases local concentrations of bioactive chemerin (Zabel et al. 2008), Zabel et al. (2008) hypothesize that Ccrl2 may influence leukocyte migration by facilitating the conversion of inactive chemerin to bioactive chemerin that subsequently binds to and directs the migration of cells expressing Cmklr1.

Inhalation exposure to ozone (O_3_), a highly reactive oxidant gas and a major air pollutant, leads to chest discomfort, cough, nose and throat irritation, and airway hyperresponsiveness (AHR) to nonspecific bronchoconstrictors such as acetyl‐*β*‐methylcholine chloride (methacholine) and histamine diphosphate (Golden et al. 1978; Kulle et al. 1985; Foster et al. 2000; Mudway and Kelly 2000). O_3_‐induced AHR contemporaneously occurs with lung injury, which is typified by lung hyperpermeability and by airway epithelial desquamation, and with lung inflammation, which is characterized, in part, by increased expression of interleukin (IL)‐6, IL‐8 [the human ortholog of mouse keratinocyte chemoattractant (KC) and macrophage inflammatory protein (MIP)‐2], and osteopontin (OPN), and by increased frequency or number of leukocytes (macrophages and neutrophils) in air spaces of the lungs (Scheel et al. 1959; Seltzer et al. 1985; Bhalla et al. 1986; Johnston et al. 2005a,b; Barreno et al. 2013; Razvi et al. 2015).

Based on data from previous investigators and our own previously published data, it is reasonable to speculate that Ccrl2 may contribute to the development of O_3_‐induced lung pathology. First, mast cells, macrophages, and neutrophils express Ccrl2 (Galligan et al. 2004; Oostendorp et al. 2004; Zabel et al. 2008; Otero et al. 2010; Del Prete et al. 2017), and published data implicate each of these cells in one or more of the various pathological sequelae induced by O_3_. For example, the use of gadolinium chloride to suppress macrophage function or nedocromil sodium to stabilize mast cell function significantly reduced the ability of O_3_ to increase lung permeability and to cause lung inflammation (Kleeberger et al. 1993b; Pendino et al. 1995). Depletion of neutrophils with cyclophosphamide attenuated O_3_‐induced lung hyperpermeability, whereas O_3_‐induced AHR was prevented when hydroxyurea was used to deplete neutrophils (O'Byrne et al. 1984; Bassett et al. 2001). Second, a recent study by Del Prete et al. (2017) reported that Ccrl2 influenced the ability of chemokine (C‐X‐C motif) receptor 2 (CXCR2), the receptor for KC and MIP‐2 (Konrad and Reutershan 2012), to promote neutrophil migration. This observation is relevant to our current study since we previously reported that CXCR2‐deficient mice had fewer bronchoalveolar lavage fluid (BALF) neutrophils (Johnston et al. 2005a). In addition, CXCR2‐deficient mice fail to develop AHR 24 hours following cessation of acute exposure to O_3_ (Johnston et al. 2005a). Third, we reported that chemerin, a ligand for Ccrl2, was increased in BALF obtained from O_3_‐exposed mice (Razvi et al. 2015). However, at present, whether increases in BALF chemerin are functionally significant following exposure to O_3_ remain unresolved.

Given these aforementioned observations, we hypothesized that Ccrl2 contributes to the development of lung pathology induced by acute exposure to O_3_. To test our hypothesis, we measured indices of lung injury (BALF protein, BALF epithelial cells, and bronchiolar epithelial injury), of lung inflammation (BALF cytokines and BALF leukocytes), and of airway responsiveness to aerosolized methacholine (respiratory system resistance) in wild‐type and mice genetically deficient in Ccrl2 (Ccrl2‐deficient mice) acutely exposed to filtered room air (air) or O_3_ [2 parts per million (ppm) for 3 hours].

## Materials and Methods

### Animals

The *Ccrl2* gene is located on the reverse strand of mouse Chromosome 9 in the second subband of the sixth major band and consists of two exons (Aken et al. 2017). Mice homozygous for a null mutation in the gene encoding *Ccrl2* (Ccrl2‐deficient mice) were generated *via* homologous recombination and characterized by Deltagen, Inc. (San Mateo, CA) (Deltagen, Inc.^2005^; Blake et al. 2017; The Jackson Laboratory, 2017). Ccrl2‐deficient mice are viable and fertile, and when compared with wild‐type mice, Ccrl2‐deficient mice display no differences in aging, behavior, blood cell differentials, body length, body mass, or serum chemistry analytes (Deltagen, Inc. 2005; Blake et al. 2017).

Cryopreserved embryos of mice heterozygous for a null mutation in the gene encoding *Ccrl2* in a C57BL/6NCrl genetic background (Charles River Laboratories, Inc., Wilmington, MA) were sent to The Jackson Laboratory (Bar Harbor, ME) from Deltagen, Inc. (personal communication with Robert Driscoll, J.D., Ph.D. of Deltagen, Inc.). At The Jackson Laboratory, the embryos of the heterozygous mice from Deltagen, Inc. were cryorecovered, and the resultant mice were backcrossed into a C57BL/6J genetic background for at least seven generations (The Jackson Laboratory, 2017). We purchased breeding pairs of Ccrl2‐deficient mice in a C57BL/6J genetic background from The Jackson Laboratory (Stock Number 005795) and housed these breeding pairs in the same room within a larger multi‐species, modified barrier animal care facility at McGovern Medical School at The University of Texas Health Science Center at Houston (Houston, TX). When at least 8 weeks of age, male and female descendants of these breeding pairs were used in the subsequently described experiments. Age‐ and gender‐matched C57BL/6J mice were purchased from The Jackson Laboratory and used as wild‐type controls. All mice were given food and water *ad libitum* and housed in the same room under previously described conditions (Razvi et al. 2015). The care and use of all animals in this study adhered to the guidelines of the National Institutes of Health (Bethesda, MD), whereas each of the experimental protocols used in this study were previously approved by the Animal Welfare Committee of The University of Texas Health Science Center at Houston (Houston, TX). The University of Texas Health Science Center at Houston has been accredited by the Association for Assessment and Accreditation of Laboratory Animal Care International since 1978.

### Protocol

Four separate cohorts of mice were used to execute the experiments in this study. However, only wild‐type mice were part of the first cohort, whereas wild‐type and Ccrl2‐deficient mice were part of cohorts two, three, and four. Mice in the first cohort were euthanized 4 or 24 hours following cessation of a 3‐hour exposure to either air or O_3_ (2 ppm). Blood and the left lung lobe were subsequently collected from each animal. All mice in the second cohort were euthanized 4 or 24 hours following cessation of a 3‐hour exposure to either air or O_3_ (2 ppm). Afterwards, blood and BALF were obtained from each animal. Mice that were part of the third cohort were euthanized 24 hours following cessation of a 3‐hour exposure to either air or O_3_ (2 ppm). Subsequently, blood was collected from each animal prior to fixing the lungs *in situ*. The lungs were then removed from each animal *en bloc*. Mice in the fourth cohort were anesthetized 24 hours following cessation of a 3‐hour exposure to either air or O_3_ (2 ppm). Quasistatic respiratory system pressure–volume (PV) curves were then generated from each mouse prior to the measurement of respiratory system responsiveness to aerosolized methacholine. Finally, data from air‐exposed, genotype‐matched mice were pooled for each outcome indicator that was assessed at both 4 and 24 hours following cessation of exposure.

### Air and O_3_ exposure

After recording the body mass of conscious wild‐type and Ccrl2‐deficient mice, the animals were individually placed into one of eight cells of a stainless steel wire mesh cage that was subsequently placed inside a powder‐coated aluminum exposure chamber with a Plexiglas^®^ door. The mice were then exposed to either air or O_3_ (2 ppm) for 3 h. Once the exposure was complete, the animals were returned to the microisolator cage that they occupied prior to the exposure. Between the time that the mice were returned to the microisolator cage and the time that the mice were anesthetized or euthanized, the mice had access to food and water *ad libitum*. In addition, immediately prior to anesthesia or euthanasia, the body mass of each mouse was again recorded. For more in‐depth details with regard to air and O_3_ exposures, please refer to a prior publication from our laboratory (Razvi et al. 2015).

### Blood collection and isolation of serum

As previously described in exhaustive detail (Razvi et al. 2015), blood was collected from the right ventricle of the heart of mice that were euthanized with an intraperitoneal injection of pentobarbital sodium (200 mg/kg; Vortech Pharmaceuticals, Ltd.; Dearborn, MI). Serum was isolated from blood by centrifugation and stored at −20°C until needed.

### Reverse transcription (RT)‐quantitative real‐time polymerase chain reactions (qPCR)

Following the collection of blood from wild‐type mice that were part of the first cohort, the left lung lobe of these animals was removed by severing the left main bronchus. Immediately after removing the left lung lobe, the lobe was snap frozen in liquid nitrogen and stored at −80°C until needed. At a later date, total ribonucleic acid (RNA) was extracted from the left lung lobe and complementary deoxyribonucleic acid was synthesized from messenger RNA (mRNA) as previously described (Razvi et al. 2015).

qPCR was performed to determine the relative abundance of *Ccrl2* mRNA in the left lung lobe using iTaq™ Universal SYBR^®^ Green Supermix (Bio‐Rad Laboratories, Inc.; Hercules, CA) and a CFX Connect™ Real‐Time PCR Detection System (Bio‐Rad Laboratories, Inc.) as *per* the instructions of the manufacturer. Using the comparative threshold cycle (C_T_) method (Livak and Schmittgen 2001), the abundance of *Ccrl2* mRNA 4 and 24 hours following cessation of exposure to O_3_ was expressed relative to the abundance of *Ccrl2* mRNA following cessation of exposure to air. All data were normalized to the abundance of hypoxanthine guanine phosphoribosyl transferase (*Hprt*) mRNA, a reference gene (Kraemer et al. 2012). Primers for *Ccrl2* and *Hprt* were purchased from Bio‐Rad Laboratories, Inc. The *Hprt* C_T_s were not different between air‐ and O_3_‐exposed mice (data not shown). Finally, melting curve analysis for both *Ccrl2* and *Hprt* primer pairs yielded a single peak, which is consistent with one product of the PCR.

### BAL

After blood was collected from the heart of mice in the second cohort, a BAL was performed on these animals and BALF was obtained. The liquid and cellular components of BALF were separated by centrifugation, and afterwards, BALF supernatant was stored at −80°C until needed for analyses. In addition, the total number of BALF cells was enumerated and differential counts of BALF cells were performed by using the cellular fraction of BALF. For each animal, the number of BALF ciliated epithelial cells, macrophages, and neutrophils were calculated by multiplying the frequency of each cell type by the total number of BALF cells. For a more complete description of the experimental details pertinent to BAL in this study, please refer to our prior work (Razvi et al. 2015).

### Cytokine and protein quantification

BALF and/or serum adiponectin, chemerin, eotaxin, hyaluronan, IL‐6, KC, MIP‐2, MIP‐3*α*, and OPN were quantified with either DuoSet^®^ enzyme‐linked immunosorbent assay (ELISA) Development Systems (R&D Systems, Inc.; Minneapolis, MN) or Quantikine^®^ ELISA Kits (R&D Systems, Inc.) as *per* the instructions of the manufacturer. BALF protein was quantified using the Bio‐Rad Protein Assay (Bio‐Rad Laboratories, Inc.) as previously described (Razvi et al. 2015).

### Lung histology

Following the collection of blood from mice in the third cohort, the lungs of these animals were fixed *in situ* at a pressure of 25 cm H_2_O with 10% phosphate‐buffered formalin (Fisher Scientific; Fair Lawn, NJ). Afterwards, the lungs and heart of each animal were removed *en bloc*, completely submerged in 10% phosphate‐buffered formalin for at least 24 hours at 4°C, dehydrated, cleared, infiltrated, and embedded in paraffin. Paraffin‐embedded sections were placed on microscope slides and stained with hematoxylin and eosin. The slides were then blindly examined by a veterinary pathologist under light microscopy to determine the bronchiolar epithelial injury score, which describes the extent of desquamation and ulceration of the airway epithelium. For specific details with regard to the procedures used to prepare the lungs for histological analysis as well as exhaustive details with regard to scoring bronchiolar epithelial injury, please refer to prior publications from our laboratory (Dahm et al. 2014; Razvi et al. 2015).

### Quasistatic respiratory system PV relationships and measurement of respiratory system responsiveness to methacholine

Twenty‐four hours following cessation of exposure to either air or O_3_, mice that were part of the fourth cohort were anesthetized with intraperitoneal injections of pentobarbital sodium (50 mg/kg; Oak Pharmaceuticals, Inc.; Lake Forest, IL) and of xylazine hydrochloride (7 mg/kg; Vedco Inc.; Saint Joseph, MO). Once the mouse was deeply anesthetized, a tracheostomy was performed on the animal, and an 18‐gauge tubing adapter (Becton, Dickinson and Company; Franklin Lakes, NJ) was inserted and secured in the lumen of the trachea. The mouse, whose chest wall remained intact, was subsequently ventilated at a frequency of 2.5 Hz, a tidal volume of 0.3 mL, and a positive end‐expiratory pressure of 3 cm H_2_O using a specialized ventilator (*flexiVent*; SCIREQ Scientific Respiratory Equipment Inc.; Montréal, Québec, Canada) (Schuessler and Bates 1995). In this study, the *flexiVent* was used to measure responses to phosphate‐buffered saline (PBS) followed by increasing doses of methacholine (Sigma‐Aldrich Co.; St. Louis, MO) for respiratory system resistance (R_RS_) using the forced oscillation technique as previously described (Razvi et al. 2015). In addition, the *flexiVent* was used to generate quasistatic respiratory system PV curves.

Prior to the measurement of responses to aerosolized PBS and to aerosolized methacholine for R_RS_, quasistatic respiratory system PV curves were generated from each mouse. To obtain quasistatic PV curves, the following procedure was executed in each mouse undergoing mechanical ventilation. First, ventilation was paused for 6 sec, and a pressure of 30 cm H_2_O was applied to the system to inflate the lungs to capacity in order to open any closed regions of the lung and to standardize lung volume history. Afterwards, ventilation was allowed to resume for at least six seconds, and then a 2.5 Hz sinusoidal forcing function was applied, whereas ventilation was paused for 1.25 sec, in order to measure R_RS_. This entire procedure was repeated at least five more times in order to ensure reproducible baseline R_RS_ values. After baseline R_RS_ was stable, the *flexiVent* delivered, over a sixteen second period, seven stepwise inspiratory volume increments that were immediately followed by seven stepwise expiratory volume increments. The first inspiratory volume increment was delivered at functional residual capacity, which is defined as lung volume at 3 cm H_2_O positive end‐expiratory pressure. Each volume increment was approximately 0.11 mL and was held for one second while airway opening pressure was measured. Subsequently, two more PV curves were generated from the same animal at 40 second intervals. From each PV curve, the following parameters of the Salazar–Knowles equation were calculated: A, an estimate of inspiratory capacity; and K, curvature of the upper portion of the expiratory limb of the PV curve (Salazar and Knowles 1964; Hartney and Robichaud 2013). Quasistatic respiratory system compliance (C_stat_) was also calculated at 5 cm H_2_O on the expiratory limb of the PV curve by fitting the Salazar–Knowles equation to each PV curve (personal communication with SCIREQ Scientific Respiratory Equipment Inc.). Finally, respiratory system hysteresis was determined by measuring the area between the inspiratory and expiratory limbs of the PV curve (Hartney and Robichaud 2013). The values of each parameter, which were derived from three different PV curves generated from each animal, were averaged to calculate a mean value for each mouse.

After PV curves were generated, we confirmed that baseline conditions were re‐established in the lungs by applying a pressure of 30 cm H_2_O to the system and subsequently measuring R_RS_ at least three times as described in the above text. Once R_RS_ was stable, responses to aerosolized PBS and to increasing doses of aerosolized methacholine (0.1 mg/mL–100 mg/mL) for R_RS_ were measured. In addition, for each dose–response curve, we calculated the ED_200_R_RS_, which is the effective dose of methacholine required to double baseline R_RS_. In this instance, baseline R_RS_ is the R_RS_ value obtained following PBS administration. For further details with regard to the generation of methacholine dose–response curves and the calculation of ED_200_R_RS_, please refer to our laboratory's prior work (Razvi et al. 2015; Elkhidir et al. 2016).

### Statistical analyses of data

The effect of genotype (wild‐type or Ccrl2‐deficient) and exposure (air or O_3_) on BALF and serum analytes, bronchiolar epithelial injury score, body mass, baseline R_RS_, and the logarithm of ED_200_R_RS_ were assessed by a two‐way analysis of variance (ANOVA) or by a Kruskal–Wallis one‐way ANOVA. The Fisher–Hayter test or the Conover–Iman test with a Bonferroni adjustment were used for *post hoc* analyses. The relative abundance of *Ccrl2* mRNA was analyzed using a Kruskal–Wallis one‐way ANOVA. Pre and postexposure body masses were compared using a Student's *t*‐test for paired samples, whereas respiratory system PV curve parameters were compared using a Student's *t*‐test for unpaired samples or a Welch's *t*‐test. Methacholine dose–response curves were analyzed using area under the curve analysis with R (Version 2.15.3) (R Core Team, 2013). All other data were analyzed using Stata 15 (StataCorp LLC; College Station, TX). Unless otherwise noted, the results are expressed as the mean ± the standard error of the mean. A *P* < 0.05 was considered significant.

## Results

### Effect of O_3_ on the relative abundance of lung *Ccrl2* mRNA in wild‐type mice

**Figure 1:**
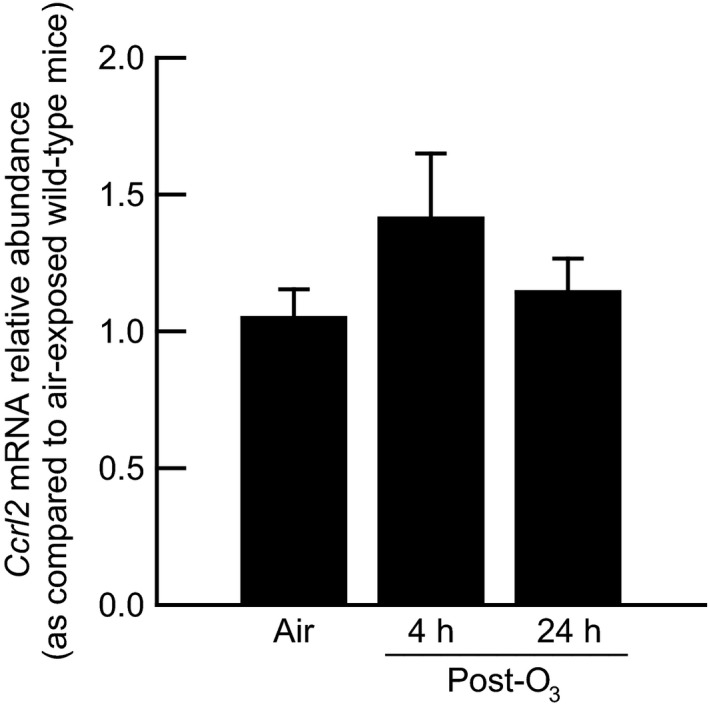
Relative abundance of chemokine (C‐C motif) receptor‐like 2 (*Ccrl2*) messenger ribonucleic acid (mRNA) in the left lung lobe of wild‐type C57BL/6 mice 4 and 24 hours following cessation of a 3‐hour exposure to ozone (O_3_; 2 parts/million). The abundance of *Ccrl2* mRNA in O_3_‐exposed mice was expressed relative to *Ccrl2* mRNA in the left lung lobe of wild‐type C57BL/6 mice 4 and 24 hours following cessation of a 3‐hour exposure to filtered room air (air). All data were normalized to the abundance of hypoxanthine guanine phosphoribosyl transferase mRNA, a reference gene, in the left lung lobe. Each value is expressed as the mean ± the standard error of the mean. *n *=* *8–10 mice in each group.

We used RT‐qPCR to determine if the relative abundance of *Ccrl2* mRNA was altered in the left lung lobe of wild‐type mice 4 and 24 hours following cessation of exposure to O_3_. When expressed relative to *Ccrl2* mRNA from the left lung lobe of air‐exposed wild‐type mice, exposure to O_3_ had no effect on the abundance of *Ccrl2* mRNA (Fig. 1).

### Effect of O_3_ and Ccrl2 deficiency on BALF and serum chemerin

Ccrl2 is one of three cell surface receptors for chemerin (Bondue et al. 2011), and exposure to O_3_ increases BALF chemerin (Razvi et al. 2015). Ccrl2 can also modulate circulating levels of chemerin in the presence of systemic inflammation (Monnier et al. 2012), a condition that is observed in mice exposed to O_3_ (Ying et al. 2016). Thus, given these observations, we determined the effect of Ccrl2 deficiency and O_3_ on BALF and serum chemerin.

**Figure 2:**
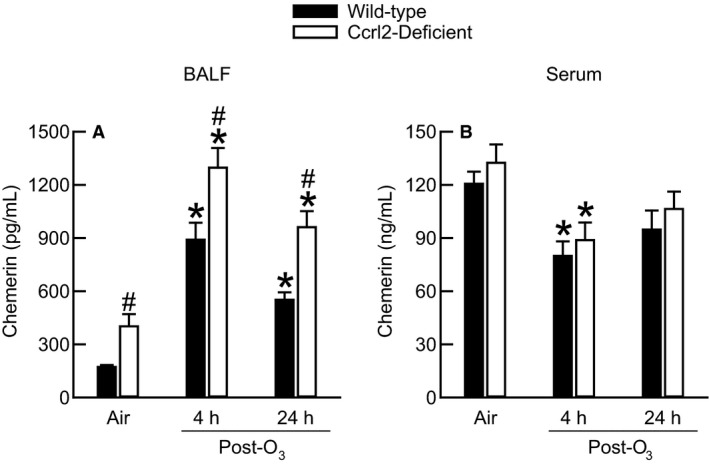
The concentration of chemerin in (A) bronchoalveolar lavage fluid (BALF) and (B) serum from wild‐type C57BL/6 mice and mice genetically deficient in chemokine (C‐C motif) receptor‐like 2 (Ccrl2‐deficient mice) 4 and 24 hours following cessation of a 3‐hour exposure to either filtered room air (air) or ozone (O_3_; 2 parts/million). Each value is expressed as the mean ± the standard error of the mean. *n *=* *8–10 mice in each group. *P <* *0.05 compared to genotype‐matched mice exposed to air. ^#^ *P *<* *0.05 compared to wild‐type mice with an identical exposure.

Chemerin was present in BALF obtained from air‐exposed wild‐type and Ccrl2‐deficient mice (Fig. 2A). However, the concentration of chemerin was significantly greater in BALF from air‐exposed Ccrl2‐deficient mice as compared to air‐exposed wild‐type mice. BALF obtained from wild‐type and Ccrl2‐deficient mice 4 or 24 hours following cessation of exposure to O_3_ contained significantly more chemerin than BALF obtained from genotype‐matched, air‐exposed controls (Fig. 2A). Nevertheless, similar to our observation in air‐exposed mice, BALF from O_3_‐exposed Ccrl2‐deficient mice contained significantly more chemerin than BALF from O_3_‐exposed wild‐type mice regardless of whether the mice were examined 4 or 24 hours following cessation of exposure.

There was no difference in serum chemerin between air‐exposed wild‐type and Ccrl2‐deficient mice (Fig. 2B). Four hours following cessation of exposure to O_3_, there was a significant reduction in the amount of chemerin present in the serum of wild‐type and Ccrl2‐deficient mice as compared to genotype‐matched, air‐exposed controls. Twenty‐four hours following cessation of exposure to O_3_, the levels of serum chemerin in wild‐type and Ccrl2‐deficient mice still remained less than those of genotype‐matched, air‐exposed controls. However, these differences were not statistically significant for either genotype.

### Effect of O_3_ and Ccrl2 deficiency on lung injury and lung inflammation

Thus far, we demonstrated that acute exposure to O_3_ had no effect on *Ccrl2* mRNA expression (Fig. 1) but did increase BALF chemerin (Fig. 2A), a ligand for Ccrl2 (Zabel et al. 2008). Ccrl2 is necessary to produce maximum injury and inflammation in response to certain stimuli (Otero et al. 2010; Douglas et al. 2013). Consequently, because inhalation of O_3_ causes lung injury and lung inflammation (Razvi et al. 2015; Elkhidir et al. 2016), we examined the potential contribution of Ccrl2 to these sequelae.

Lung hyperpermeability and airway epithelial desquamation are two features of O_3_‐induced lung injury (Scheel et al. 1959; Bhalla et al. 1986). Disruption of the alveolar‐capillary membrane induced by inhalation exposure to O_3_ causes serum proteins to diffuse to air spaces, and the accumulation of protein in BALF is a useful indicator to assess lung permeability following exposure to O_3_ (Alpert et al. 1971; Hu et al. 1982). Thus, to evaluate O_3_‐induced lung injury in this study, we measured BALF protein, enumerated the number of ciliated epithelial cells in BALF, and histologically scored bronchiolar epithelial injury.

**Figure 3:**
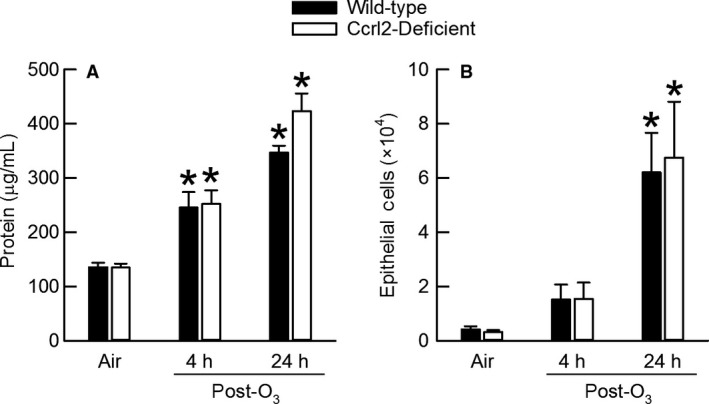
(A) The concentration of protein and (B) the number of epithelial cells in bronchoalveolar lavage fluid from wild‐type C57BL/6 mice and mice genetically deficient in chemokine (C‐C motif) receptor‐like 2 (Ccrl2‐deficient mice) 4 and 24 hours following cessation of a 3‐hour exposure to either filtered room air (air) or ozone (O_3_; 2 parts/million). Each value is expressed as the mean ± the standard error of the mean. *n *=* *8–10 mice in each group. *P <* *0.05 compared to genotype‐matched mice exposed to air.

There was no difference in the concentration of BALF protein between air‐exposed wild‐type and Ccrl2‐deficient mice (Fig. 3A). Regardless of whether wild‐type or Ccrl2‐deficient mice were examined 4 or 24 hours following cessation of exposure to O_3_, O_3_ caused a significant increase in BALF protein as compared to genotype‐matched, air‐exposed controls. Nevertheless, no genotype‐related differences in BALF protein existed at any time interval following cessation of O_3_ exposure. The number of BALF epithelial cells was not different between wild‐type and Ccrl2‐deficient mice following cessation of air exposure (Fig. 3B). As compared to genotype‐matched, air‐exposed controls, O_3_ significantly increased BALF epithelial cells in wild‐type and Ccrl2‐deficient mice 24 hours following cessation of exposure to O_3_. However, no genotype‐related difference in BALF epithelial cells existed after O_3_ exposure.

**Figure 4:**
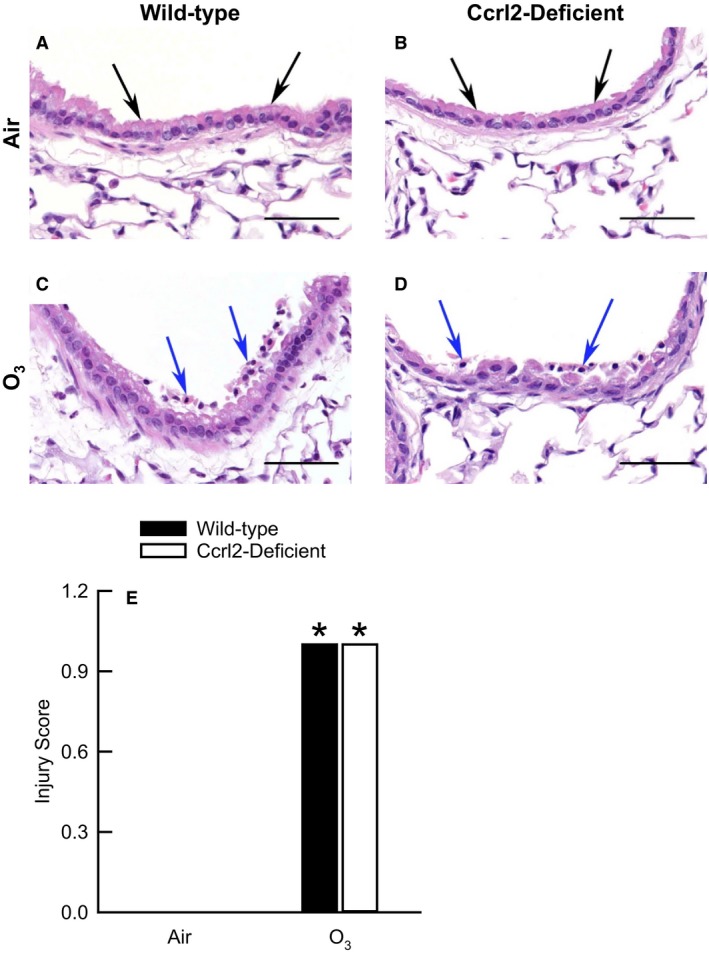
(A−D) Representative light photomicrographs of hematoxylin‐ and eosin‐stained lung sections and (E) bronchiolar epithelial injury scores from wild‐type C57BL/6 mice and mice genetically deficient in chemokine (C‐C motif) receptor‐like 2 (Ccrl2‐deficient mice) 24  hours following cessation of a 3‐hour exposure to either filtered room air (air) or ozone (O_3_; 2 parts/million). A and B are lung sections from air‐exposed wild‐type and Ccrl2‐deficient mice, respectively. C and D are lung sections from O_3_‐exposed wild‐type and Ccrl2‐deficient mice, respectively. The black arrows in A and B are directed at bronchiolar epithelial cells that appear normal and are attached to the basement membrane, whereas the blue arrows in C and D are directed at detached bronchiolar epithelial cells. In D, the detached, degenerate epithelial cells are associated with flattening and erosion of the underlying mucosa. In A−D, the images have been magnified with a 40 ×  objective lens while each of the scale bars in A−D represent 50 *μ*m. In E, each value is expressed as the mean ± the standard error of the mean. *n *=* *8 mice in each group. *P <* *0.05 compared to genotype‐matched mice exposed to air.

Because the number of BALF epithelial cells was increased in wild‐type and Ccrl2‐deficient mice 24 hours following cessation of exposure to O_3_ (Fig. 3B), we semi‐quantitatively scored bronchiolar epithelial injury in hematoxylin‐ and eosin‐stained lungs sections that were prepared from formalin‐fixed and paraffin‐embedded lungs obtained from wild‐type and Ccrl2‐deficient mice 24 hours following cessation of exposure to air or O_3_ (Fig. 4). Wild‐type and Ccrl2‐deficient mice exposed to air exhibited no significant lesions (Fig. 4A, B, and E). The epithelial cells in these mice were typically columnar and appeared normal and attached to the subjacent basement membrane. However, lungs from O_3_‐exposed mice consistently exhibited minimal, widespread, multifocal, yet significant injury to the bronchiolar epithelium (Fig. 4C, D, and E). This injury was characterized by the presence of multifocal groups of detached epithelial cells in the bronchiolar lumen. The detached epithelial cells were often associated with focal areas of bronchiolar epithelial erosion and flattening of the underlying epithelial cells. Nevertheless, no genotype‐related difference in bronchiolar epithelial injury existed following cessation of O_3_ exposure.

**Figure 5:**
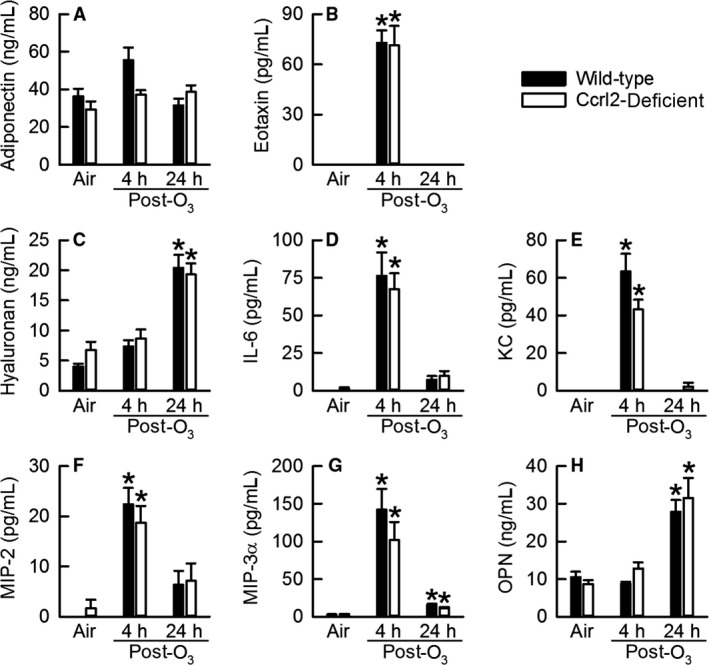
The concentration of (A) adiponectin, (B) eotaxin, (C) hyaluronan, (D) interleukin (IL)‐6, (E) keratinocyte chemoattractant (KC), (F) macrophage inflammatory protein (MIP)‐2, (G) MIP‐3*α*, and (H) osteopontin (OPN) in bronchoalveolar lavage from wild‐type C57BL/6 mice and mice genetically deficient in chemokine (C‐C motif) receptor‐like 2 (Ccrl2‐deficient mice) 4 and 24 hours following cessation of a 3‐hour exposure to either filtered room air (air) or ozone (O_3_; 2 parts/million). Each value is expressed as the mean ± the standard error of the mean. *n *=* *8–10 mice in each group. *P <* *0.05 compared to genotype‐matched mice exposed to air.

We also measured the concentration of cytokines in BALF that have been previously shown to contribute to various sequelae of lung pathology induced by acute exposure to O_3_, including AHR (adiponectin, hyaluronan, KC, MIP‐2, and OPN), airway epithelial cell desquamation (IL‐6, KC, and MIP‐2), lung hyperpermeability (adiponectin), and macrophage and/or neutrophil migration to air spaces (adiponectin, hyaluronan, IL‐6, KC, MIP‐2, and OPN) (Johnston et al. 2005a,b; Lang et al. 2008; Garantziotis et al. 2009, 2016; Zhu et al. 2010; Barreno et al. 2013). Although eotaxin and MIP‐3*α* have not yet been specifically implicated in any of the aforementioned sequelae of O_3_‐induced lung pathology, we measured the levels of these cytokines since they have been previously demonstrated to be expressed in the lung following acute exposure to O_3_ (Johnston et al. 2007; Williams et al. 2008). In air‐exposed mice, there were no genotype‐related differences in any of the cytokines examined (Fig. 5). However, with the exception of adiponectin, the appearance of these cytokines in BALF following cessation of O_3_ exposure took two different courses depending on the time interval examined. For eotaxin, IL‐6, KC, MIP‐2, and MIP‐3*α*, the levels of each of these cytokines were significantly greater than genotype‐matched, air‐exposed controls at four hours following cessation of exposure to O_3_, but with the exception of MIP‐3*α*, these cytokines were barely detectable in BALF at 24 hours following cessation of exposure to O_3_ (Figure 5B, D, E, F, and G). In wild‐type and Ccrl2‐deficient mice, BALF hyaluronan and OPN were not significantly different from genotype‐matched, air‐exposed controls at four hours following cessation of O_3_ exposure but were significantly greater than genotype‐matched, air‐exposed controls at 24 hours following cessation of exposure to O_3_ (Fig. 5C and H). As compared to wild‐type mice exposed to air, O_3_ increased BALF adiponectin 4 hours following cessation of O_3_ exposure (Fig. 5A). However, this increase was not statistically significant (*P *=* *0.09). Finally, there were no genotype‐related differences in any of these cytokines 4 or 24 hours following cessation of exposure to O_3_.

**Figure 6:**
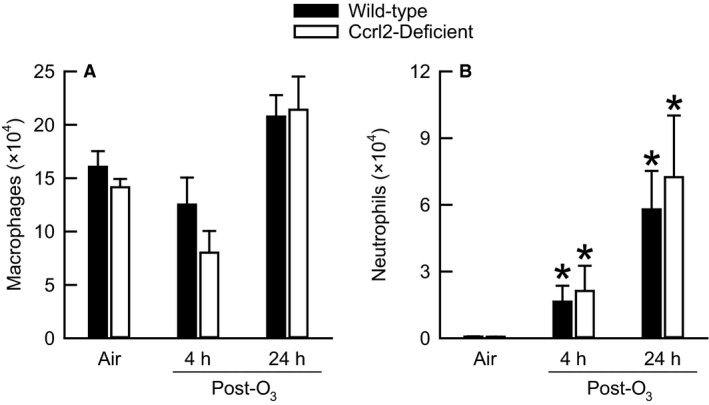
The number of (A) macrophages and (B) neutrophils in bronchoalveolar lavage fluid from wild‐type C57BL/6 mice and mice genetically deficient in chemokine (C‐C motif) receptor‐like 2 (Ccrl2‐deficient mice) 4 and 24 hours following cessation of a 3‐h exposure to either filtered room air (air) or ozone (O_3_; 2 parts/million). Each value is expressed as the mean ± the standard error of the mean. *n *=* *8–10 mice in each group. *P <* *0.05 compared to genotype‐matched mice exposed to air.

Macrophage and neutrophil migration to air spaces is commonly observed after exposure to O_3_ (Razvi et al. 2015). In addition, both macrophages and neutrophils contribute to O_3_‐induced lung pathology (O'Byrne et al. 1984; Pendino et al. 1995). Consequently, we enumerated the number of macrophages and neutrophils in BALF following cessation of exposure to O_3_ (Fig. 6). There were no differences in the number of macrophages or neutrophils between air‐exposed wild‐type and Ccrl2‐deficient mice (Fig. 6). Four and twenty‐four hours following cessation of exposure to O_3_, the number of BALF macrophages in wild‐type and Ccrl2‐deficient mice was not significantly different from genotype‐matched, air‐exposed controls (Fig. 6A). In mice of both genotypes, the number of BALF neutrophils was significantly greater in O_3_‐ as compared to air‐exposed mice regardless of the time interval that the cells were enumerated (Fig. 6B). Nevertheless, there were no genotype‐related differences in the number of macrophages or neutrophils at any time following cessation of exposure to O_3_.

### Effect of Ccrl2 deficiency on quasistatic respiratory system PV relationships in air‐exposed mice

Chemerin‐15, a synthetic peptide, signals *via* Cmklr1 to elicit many of the same biological effects as chemerin (Cash et al. 2008). Cash et al. (2014) reported that chemerin‐15 alters collagen deposition in injured skin. Since chemerin‐15 and chemerin signal *via* Cmklr1 (Cash et al. 2008; Bondue et al. 2011), it is plausible that chemerin‐Cmklr1 signaling modifies collagen deposition in skin as well as in other organs, including the lungs, a phenomenon that would significantly impact the quasistatic elastic properties of the respiratory system. Because Ccrl2 influences the biological effects of Cmklr1 (Monnier et al. 2012), it is also reasonable to suspect that Ccrl2 may be involved in collagen deposition, and thus, modulate the quasistatic elastic properties of the respiratory system. To that end, we generated and subsequently examined quasistatic respiratory system PV curves from air‐exposed wild‐type and Ccrl2‐deficient mice.

**Figure 7:**
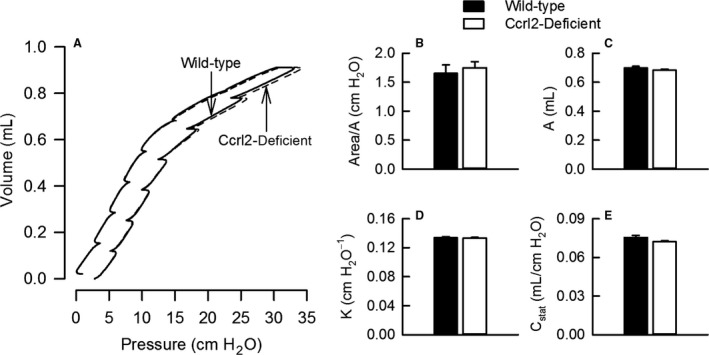
(A) Quasistatic respiratory system pressure–volume (PV) curves, (B) area (hysteresis) of PV curves normalized for A, an estimate of inspiratory capacity, (C) A, (D) K, the parameter from the Salazar–Knowles equation reflecting the curvature of the upper portion of the expiratory limb of the PV curve, and (E) C_stat_, static compliance of the respiratory system. PV curves were initiated from functional residual capacity, which is defined as lung volume at 3 cm H_2_O positive end‐expiratory pressure, and generated by subsequent volume displacement. The PV curves and associated parameters were obtained from wild‐type C57BL/6 mice and mice genetically deficient in chemokine (C‐C motif) receptor‐like 2 (Ccrl2‐deficient mice) 24 hours following cessation of a 3‐hour exposure to filtered room air. In B − E, each value is expressed as the mean ± the standard error of the mean. *n *=* *10–12 mice in each group.

As shown in Figure 7A, the respiratory system PV curves from air‐exposed wild‐type and Ccrl2‐deficient mice were superimposed, which suggested that the quasistatic elastic properties of the respiratory system were not different between wild‐type and Ccrl2‐deficient mice. To quantitatively confirm this observation, we calculated the hysteresis of the respiratory system PV curves by normalizing the area enclosed by the PV curve by A, an estimate of inspiratory capacity. As shown in Figure 7B, there was no effect of genotype on respiratory system hysteresis (Area/A) or A (Fig. 7C). Finally, K and C_stat_ were also unaffected by Ccrl2 deficiency (Fig. 7D and E). These data demonstrate that Ccrl2 does not modulate the quasistatic elastic properties of the respiratory system.

### Effect of O_3_ and Ccrl2 deficiency on body mass and respiratory system responsiveness to methacholine

Immediately prior to O_3_ exposure, the body masses of wild‐type and Ccrl2‐deficient mice were not different from each other (Table 1). Exposure to O_3_ caused a significant decrease in the body masses of wild‐type and Ccrl2‐deficient mice, which is consistent with the ability of inhaled O_3_ to induce cachexia (Last et al. 2005).

**Table 1:** Pre and postexposure body mass, respiratory system resistance at baseline, and effective dose of methacholine necessary to cause a 200% increase in respiratory system resistance at baseline for wild‐type C57BL/6 and Ccrl2‐deficient mice exposed to filtered room air or ozone

Genotype (Exposure)	Body Mass (g)		R_RS_ (cm H_2_O/ml/s)	ED_200_R_RS_ (mg/mL) (95% Confidence Interval)
	PreExposure	PostExposure		
Wild‐type (Air)	26.0 ± 0.9	25.8 ± 0.9	0.62 ± 0.02	2.8 (1.9–4.2)
Ccrl2‐Deficient (Air)	24.7 ± 0.8	24.5 ± 0.8	0.60 ± 0.02	2.5 (1.8–3.4)
Wild‐type (O_3_)	24.6 ± 1.0	22.4 ± 1.2^a^	0.66 ± 0.02	1.7 (1.4–2.0)
Ccrl2‐Deficient (O_3_)	25.1 ± 1.4	23.2 ± 1.5^a^	0.63 ± 0.04	1.4 (0.9–2.3)

The results are expressed as the mean ± the standard error of the mean for body mass and respiratory system resistance at baseline (R_RS_) or mean and 95% confidence interval for effective dose of methacholine necessary to cause a 200% increase in R_RS_ at baseline (ED_200_R_RS_). Measurements of preexposure body mass were made immediately prior to exposure to filtered room air (air) or ozone (O_3_; 2 parts/million) for 3 hours, whereas measurements of postexposure body mass were made in the same animals 24 hours following cessation of a 3‐hour exposure to air or O_3_. Measurements of R_RS_ at baseline were made following administration of phosphate‐buffered saline. R_RS_ at baseline and ED_200_R_RS_ were measured or calculated, respectively, 24 hours following cessation of a 3‐hour exposure to either air or O_3_. *n *=* *10–13 mice in each group.

a *P *<* *0.05 compared to preexposure body mass of genotype‐matched mice.

**Figure 8:**
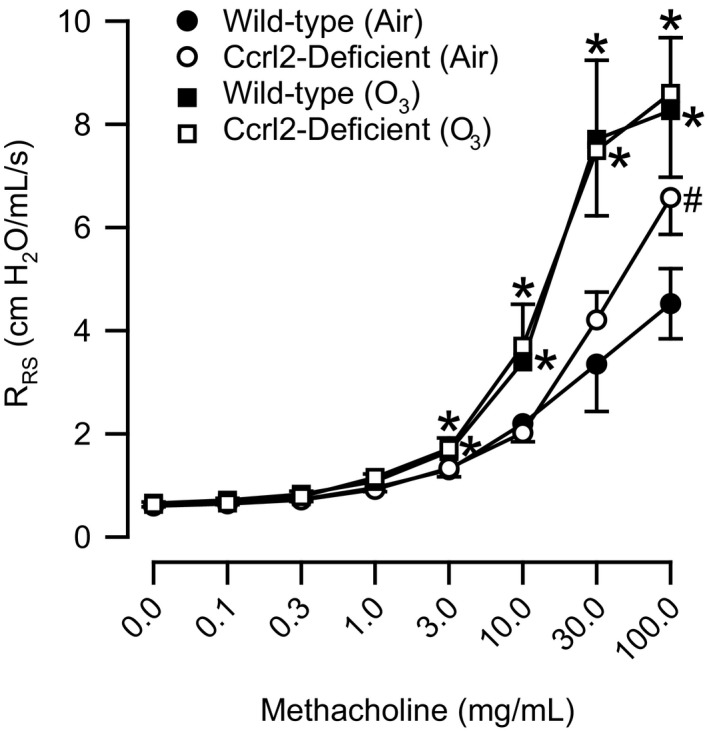
Responses to aerosolized acetyl‐*β*‐methylcholine chloride (methacholine) for respiratory system resistance (R_RS_) in wild‐type C57BL/6 mice and mice genetically deficient in chemokine (C‐C motif) receptor‐like 2 (Ccrl2‐deficient mice) 24 hours following cessation of a 3‐hour exposure to either filtered room air (air) or ozone (O_3_; 2 parts/million). Each value is expressed as the mean ± the standard error of the mean. *n *=* *10–13 mice in each group. *P <* *0.05 compared to genotyped‐matched mice exposed to air. ^#^ *P *<* *0.05 compared to wild‐type mice with an identical exposure.

Twenty‐four hours following cessation of exposure to air, baseline R_RS_ was not different between wild‐type and Ccrl2‐deficient mice (Table 1). Methacholine significantly increased R_RS_ in air‐exposed mice. However, with the exception of the response to 100 mg/mL of methacholine, which was significantly greater in Ccrl2‐deficient as compared to wild‐type mice, responses to all other concentrations of methacholine for R_RS_ in air‐exposed mice were not different between genotypes (Fig. 8). The ED_200_R_RS_ was unaffected by genotype in air‐exposed mice (Table 1). When compared to genotype‐matched, air‐exposed controls, O_3_ increased baseline R_RS_ in wild‐type and Ccrl2‐deficient mice. However, these increases were not significant for either genotype (Table 1). Similar to our observation in air‐exposed mice, methacholine increased R_RS_ in O_3_‐exposed wild‐type and Ccrl2‐deficient mice. At all methacholine concentrations greater than or equal to 3 mg/mL, O_3_ significantly increased responses to methacholine for R_RS_ when compared to the same responses in genotype‐matched, air‐exposed controls (Fig. 8). Nevertheless, there was no effect of genotype on responsiveness to methacholine following O_3_ exposure. Finally, although the ED_200_R_RS_ was decreased in both O_3_‐exposed wild‐type and Ccrl2‐deficient mice when compared to genotype‐matched, air‐exposed controls, these decreases were not statistically significant for mice of either genotype.

## Discussion

As mentioned in the Introduction, there a number of observations that suggest a potential role for Ccrl2 in the development of O_3_‐induced lung pathology. Our data, however, demonstrate that Ccrl2 deficiency has no effect on the development of lung injury, lung inflammation, or AHR four and/or 24 hours following cessation of a 3‐hour exposure to O_3_ (2 ppm) (Fig. 3, 4, 5, 6 and Fig. 8). Nevertheless, we do demonstrate that Ccrl2 modulates the levels of chemerin in the epithelial lining fluid of the lungs following air or O_3_ exposure (Fig. 2).

Injurious stimuli are potent inducers of Ccrl2 expression. First, lipopolysaccharide (LPS) increases expression of Ccrl2 in astrocytes, bone marrow‐derived myeloid dendritic cells and neutrophils, endothelial cells, microglia, and peritoneal macrophages (Shimada et al. 1998; Zuurman et al. 2003; Zabel et al. 2008; Otero et al. 2010; Monnier et al. 2012; Del Prete et al. 2017). Second, LPS in combination with transforming growth factor *β*1 and interferon gamma (IFN‐*γ*) increase Ccrl2 expression in astrocytes (Hamby et al. 2012). Third, the presence of experimental autoimmune encephalomyelitis in mice induces *Ccrl2* mRNA expression in central nervous system mononuclear cells (Mazzon et al. 2016). Fourth, Oostendorp et al. (2004) reported that Ccrl2 expression was rapidly induced in bronchial epithelium and Mac‐3^+^ lung macrophages following antigen sensitization and challenge. Given the aforementioned observations that Ccrl2 expression is up‐regulated in a number of cells following injury, we were quite surprised that acute exposure to O_3_ did not significantly increase the relative abundance of *Ccrl2* mRNA in wild‐type mice either 4 or 24 hours following cessation of exposure (Fig. 1). Nevertheless, there are potential scenarios that could explain these observations. First, *Ccrl2* mRNA expression may have been significantly increased either before or after the time intervals at which our measurements were made. For example, *Ccrl2* mRNA expression peaked in endothelial cells two hours following treatment with IFN‐*γ*, LPS, and tumor necrosis factor‐*α* and then declined thereafter (Monnier et al. 2012). Second, O_3_ and/or the various ozonation products generated from the interaction of O_3_ with lipids and proteins in the epithelial lining fluid of the lungs may not have been sufficient stimuli to increase *Ccrl2* mRNA expression in the lungs. Finally, although not a scenario to explain the inability of O_3_ to increase *Ccrl2* mRNA expression 4 and 24 hours following cessation of exposure, it is certainly plausible that Ccrl2 protein expression may have increased following cessation of O_3_ exposure in the absence of an increase in *Ccrl2* mRNA. Consequently, in the future, it may be necessary to quantify Ccrl2 protein expression in response to injurious stimuli when no change in *Ccrl2* mRNA is observed.

Although we did not observe a change in the relative abundance of *Ccrl2* mRNA following O_3_ exposure (Fig. 1), we did observe robust increases in BALF chemerin in wild‐type and Ccrl2‐deficient mice 4 and 24 hours following cessation of exposure to O_3_ (Fig. 2A), which is consistent with a previous observation from our laboratory (Razvi et al. 2015). Thus, O_3_ can be added to the list of inflammatory stimuli that increase expression of chemerin, including antigen sensitization and challenge, cigarette smoke, IL‐1*β*, LPS, and obesity (Conde et al. 2011; Demoor et al. 2011; Monnier et al. 2012; Dahm et al. 2014). In BALF, the levels of chemerin were significantly greater in Ccrl2‐deficient as compared to wild‐type mice regardless of whether the mice were exposed to air or O_3_ (Fig. 2A). Similar to our observations with BALF chemerin in Ccrl2‐deficient mice, Monnier et al. (2012) previously reported that serum levels of chemerin were greater in Ccrl2‐deficient as compared to wild‐type mice that were injected intraperitoneally with either saline or LPS. However, we observed no effect of Ccrl2 deficiency on serum chemerin following air or O_3_ exposure (Fig. 2B). In fact, when compared to genotype‐matched, air‐exposed mice, serum chemerin was significantly decreased four hours after exposure to O_3_. Lung permeability increases following O_3_ exposure, which is demonstrated by an increase in BALF protein (Fig. 3A). Thus, the reduction in serum chemerin 4 h following cessation of exposure to O_3_ may be a result of chemerin diffusing from blood to air spaces at a greater rate than it can be replenished in the circulation. Nevertheless, our data with regard to genotype‐related differences in BALF chemerin are consistent with the previous observation that Ccrl2 concentrates chemerin on the cell surface (Monnier et al. 2012). The bronchial epithelium is an abundant source of Ccrl2 in the absence of any inciting stimulus and following antigen sensitization and challenge (Oostendorp et al. 2004), and since Ccrl2 concentrates chemerin on the cell surface (Monnier et al. 2012), the loss of Ccrl2 likely prevents chemerin sequestration, which results in more chemerin in the epithelial lining fluid of the lung. The absence of any genotype‐related difference in serum chemerin suggest that Ccrl2 is not a significant source of chemerin sequestration in the blood, and/or alternatively, one or more of the other cell surface receptors for chemerin (Cmklr1 and Gpr1) bind more chemerin in the blood in the absence of Ccrl2 such that no difference in serum chemerin exists between Ccrl2‐deficient and wild‐type mice. Taken together, these data demonstrate that Ccrl2 modulates chemerin levels in the epithelial lining fluid of the lungs.

O_3_ caused lung injury in wild‐type and Ccrl2‐deficient mice, which was demonstrated by an increase in BALF protein and by airway epithelial desquamation (Fig. 3 and Fig. 4). However, Ccrl2 deficiency had no effect on either of these outcome indicators following exposure to O_3_. We and others have previously demonstrated that adiponectin, macrophages, and neutrophils contribute to the development of O_3_‐induced lung hyperpermeability, whereas IL‐6 and CXCR2, the receptor for KC and MIP‐2, promote epithelial cell desquamation following O_3_ exposure (Pendino et al. 1995; Bassett et al. 2001; Johnston et al. 2005a; Lang et al. 2008; Zhu et al. 2010; Konrad and Reutershan 2012). There were no genotype‐related differences in BALF adiponectin, IL‐6, KC, MIP‐2, macrophages, or neutrophils following O_3_ exposure (Fig. 5A, D, E, and F and Fig. 6), and since each of these cytokines or cells are involved in promoting O_3_‐induced lung injury, it not surprising that we observed no genotype‐related differences in BALF protein or airway epithelial desquamation following cessation of exposure to O_3_. From these data, we can conclude that Ccrl2 does not contribute to the development of lung injury following acute exposure to O_3_.

A recent study by Del Prete et al. (2017) demonstrated that Ccrl2 is necessary for maximum CXCR2‐induced neutrophil migration. We previously reported that CXCR2 is responsible for the recruitment of the majority of neutrophils to air spaces following acute exposure to O_3_ (Johnston et al. 2005a). We also made the same observation for OPN, an acidic glycoprotein (Barreno et al. 2013). In fact, both CXCR2‐deficient and OPN‐deficient mice have a similar reduction in neutrophil recruitment to air spaces following O_3_ exposure (Johnston et al. 2005a; Barreno et al. 2013), which suggests a common pathway may exist for CXCR2‐ and OPN‐induced neutrophil migration. Indeed, Singh et al. (2017) recently reported that OPN was necessary for maximal CXCR2‐induced neutrophil recruitment. However, from our data, it is not possible to determine the precise role of Ccrl2 or OPN in CXCR2‐induced neutrophil recruitment following O_3_ exposure. However, we can speculate about a number of possibilities given the fact that neither CXCR2 expression nor BALF KC, MIP‐2, or OPN are affected by Ccrl2 deficiency [Fig. 5E, F, and H and (Del Prete et al. 2017)]. First, if Ccrl2 was absolutely necessary for CXCR2‐induced neutrophil recruitment following cessation of O_3_ exposure, regardless of whether OPN was also required for CXCR2‐induced neutrophil recruitment in our model, we would expect BALF neutrophils to be significantly reduced in Ccrl2‐deficient as compared to wild‐type mice. Second, it is possible that Ccrl2 is essential for CXCR2‐induced neutrophil migration after cessation of O_3_ exposure, but this effect is masked by OPN‐mediated neutrophil migration that is CXCR2‐independent. For example, Schneider et al. (2010) reported that OPN can facilitate neutrophil chemotaxis by engaging at least two of its cell surface receptors: CD44 and integrin *α*_V_ *β*_3_ (Denhardt et al. 2001). Thus, if Ccrl2 is necessary for CXCR2‐induced neutrophil migration in O_3_‐exposed mice, OPN engagement of CD44 and *α*_V_ *β*_3_ in Ccrl2‐deficient mice could compensate for the loss of Ccrl2‐CXCR2‐dependent neutrophil migration, which would ultimately result in normal neutrophil recruitment in Ccrl2‐deficient mice. Third, Ccrl2 may be completely unnecessary for CXCR2‐induced neutrophil migration following cessation of O_3_ exposure, regardless of whether OPN was also essential for CXCR2‐dependent neutrophil migration in our model. This scenario would also result in no defect in neutrophil migration in Ccrl2‐deficient mice. Based on the observation that BALF neutrophils were not different between wild‐type and Ccrl2‐deficient mice following exposure to O_3_ (Fig. 6B), the second and third scenarios described above are the most probable.

AHR to nonspecific bronchoconstrictors, including histamine, methacholine, and serotonin, is commonly observed following acute exposure to O_3_ (Golden et al. 1978; Foster et al. 2000; Lu et al. 2006; Barreno et al. 2013). Consistent with these results, wild‐type and Ccrl2‐deficient mice exhibited AHR to inhaled methacholine 24 hours following cessation of exposure to O_3_ (Fig. 8). However, there was no genotype‐related difference in responsiveness to methacholine following O_3_ exposure. We and others have previously demonstrated that adiponectin, hyaluronan, KC, MIP‐2, and OPN are necessary for the development of O_3_‐induced AHR (Johnston et al. 2005a; Garantziotis et al. 2009, 2016; Zhu et al. 2010; Barreno et al. 2013). Following O_3_ exposure, however, there were no differences in any of these cytokines in BALF obtained from wild‐type and Ccrl2‐deficient mice (Fig. 5A, C, E, F, and H). Consequently, it was not unexpected that responsiveness to methacholine following cessation of exposure to O_3_ was not different between wild‐type and Ccrl2‐deficient mice. To the best of our knowledge, there has been only one prior study that investigated the role of Ccrl2 in the development of AHR. Otero et al. (2010) examined airway responsiveness to methacholine in wild‐type and Ccrl2‐deficient mice following antigen sensitization and challenge. Although the investigators reported that airway responsiveness following antigen sensitization and challenge was not different between wild‐type and Ccrl2‐deficient mice, the investigators did demonstrate that BALF IL‐4, IL‐5, eosinophils, and lymphocytes were significantly lower in Ccrl2‐deficient as compared to wild‐type mice (Otero et al. 2010). Given the different mechanisms by which antigen sensitization and challenge and O_3_ lead to lung inflammation, the ability of Ccrl2 to contribute to an inflammatory response may be stimulus‐specific.

Because cells that express Ccrl2 have been previously implicated in O_3_‐induced lung pathology (O'Byrne et al. 1984; Kleeberger et al. 1993b; Pendino et al. 1995; Bassett et al. 2001; Galligan et al. 2004; Oostendorp et al. 2004; Zabel et al. 2008; Otero et al. 2010; Del Prete et al. 2017), we were quite surprised that Ccrl2 deficiency failed to lessen the severity of the sequelae induced by inhaled O_3_. As mentioned above, the inability of Ccrl2 deficiency to reduce injury and inflammation may depend on the injurious stimulus. However, there are other possibilities. First, in addition to Ccrl2, chemerin is a ligand for Cmklr1 and Gpr1 (Bondue et al. 2011). Cmklr1 signaling results in both pro‐ and anti‐inflammatory effects (Cash et al. 2008; Demoor et al. 2011; Provoost et al. 2016). Thus, in the absence of Ccrl2, the availability of chemerin to Cmklr1 may increase and perhaps result in proinflammatory effects that offset any reduction in inflammation caused by Ccrl2 deficiency. Second, we and others have demonstrated that adiponectin, type I IL‐1 receptor, and IL‐6 elicit diverse pulmonary responses to O_3_ that depend on the duration of exposure to the toxic gas (Johnston et al. 2005b, 2007; Zhu et al. 2010; Kasahara et al. 2012). These data are consistent with a report from Kleeberger et al. (1993a), which demonstrates that separate genetic loci contribute to pulmonary responses induced by acute as compared to prolonged O_3_ exposure. The contribution of mast cells, which express Ccrl2, to O_3_‐induced lung injury are different for acute as compared to prolonged O_3_ exposure (Kleeberger et al. 1993b, 2001; Zabel et al. 2008). As a consequence, the contribution of Ccrl2 to the development of lung injury and lung inflammation following subacute or chronic O_3_ exposure may be different than following acute O_3_ exposure.

## Conclusions

In summary, our data demonstrate that genetic deficiency of Ccrl2, one of three seven‐transmembrane domain receptors for the nonchemokine chemoattractant, chemerin, had no effect on the development of lung injury, lung inflammation, or AHR following cessation of acute exposure to O_3_. Nevertheless, our data do demonstrate that Ccrl2 modulates the levels of chemerin in the epithelial lining fluid of the lungs in the absence of any inciting stimulus and after cessation of acute O_3_ exposure.

## Conflict of Interest

No conflicts of interest, financial or otherwise, are declared by the authors.
